# Toll-like receptor-mediated immune imbalance in asthma: controversies, breakthroughs, and future directions

**DOI:** 10.3389/fimmu.2025.1605185

**Published:** 2025-07-02

**Authors:** Xinyi Xu, Ruihan Yu, Zhuoqun Yang, Chenyu Li, Huabao Xiong, Chunxia Li

**Affiliations:** Institute of Immunology and Molecular Medicine, Jining Medical University, Jining, China

**Keywords:** toll-like receptors, toll-like receptors signaling, asthma, immune cells, inflammation, cytokines, therapeutic strategies

## Abstract

As a chronic inflammatory illness of the respiratory system, asthma occurs due to various factors and is characterized by a T helper 2 (Th2)-skewed immune response, airway hyperresponsiveness, and reversible airflow obstruction. Toll-like receptors (TLRs) perform a “double-edged sword” function in asthma-related immunological dysregulation by recognizing damage-associated molecular patterns and pathogen-associated molecular patterns. In turn, the activation of some TLRs stimulates epithelial cells to release inflammatory cytokines, exacerbating Th2-driven inflammation and contributing to airway remodeling. Certain TLR signals help inhibit allergic responses by inducing type I interferon or regulatory T cells. The TLR family comprises 10 members, each responsible for recognizing the distinct molecular structure of multiple microbial sources. Variations in environmental microbial exposure duration and host genetic background contribute to the complexity of the TLR signaling network during asthma development. In recent years, therapeutic strategies targeting TLRs have shown potential for asthma treatment. However, a comprehensive review of TLRs in asthma is lacking. Therefore, this review sought to examine the functional mechanisms of TLRs and associated signaling cascades in asthma, and explore novel prevention and treatment approaches centered on TLRs modulation.

## 1 Introduction

Asthma, a prevalent respiratory condition affecting over 330 million individuals worldwide (1, 2), is a fundamentally heterogeneous disease. This heterogeneity manifests as multiple distinct phenotypes, which are clinically defined by variations in presenting features, triggering factors, patterns of airway inflammation, and physiological or pathological characteristics (3). Underpinning these phenotypes is a complex and persistent inflammatory process within the airways. Key features of inflammation in asthma include airway hyperresponsiveness, eosinophilic infiltration, excessive mucus production, reversible airflow limitation, structural airway remodeling, and goblet cell hyperplasia (1). The innate immune system plays a pivotal role against this backdrop of complex airway pathology. Toll-like receptors (TLRs) represent a category of pattern recognition receptors that detect damage-associated molecular patterns (DAMPs) and pathogen-associated molecular patterns (PAMPs), triggering innate immune responses (4). TLRs function as a vital connection between adaptive and innate immunity by modulating the activation of essential cytokines and antigen-presenting cells (5). They play a crucial part in numerous diseases, including atherosclerosis, acute lymphoblastic leukemia, Parkinson’s disease, sepsis, cancer, and autoimmune disorders (6–9). Recent studies show that TLRs contribute to the onset and exacerbation of asthma. The purpose of this review is to investigate the function of TLRs in respiratory inflammation, their contribution to the pathogenesis of asthma, and new developments in targeting TLRs for asthma treatment.

## 2 Pathogenesis of asthma

Asthma is a heterogeneous disease with multiple phenotypes, including allergic, non-allergic, late-onset, asthma with fixed airflow limitation, and asthma with obesity, which are shaped by differences in age of onset, clinical features, triggers, and inflammatory mechanisms. The pathophysiology of asthma involves activation of both innate and adaptive immune responses, leading to chronic airway inflammation. This inflammation results in airway edema, mucus hypersecretion, and remodeling, characterized by subepithelial fibrosis, basement membrane thickening, smooth muscle hypertrophy, angiogenesis, and mucous gland hyperplasia. These structural changes are driven, in part, by Th1 (type1) and Th2 (type 2) cell-mediated immune responses, contributing to persistent airway dysfunction (10). In typical allergen-induced asthma, the pathogenesis is frequently characterized by an inflammatory environment dominated by the type 2 immune response. Under normal conditions, a balance exists between type 1 and type 2 cells. However, when harmful agents, such as allergens, viruses, and bacteria, infiltrate the respiratory tract, they initially trigger airway epithelial cells to release epithelial-derived cytokines, including interleukin-33 (IL-33), IL-25, and thymic stromal lymphopoietin. These cytokines act as early warning signals for potential epithelial and endothelial damage. Concurrently, these agents stimulate antigen-presenting cells, especially dendritic cells, to perform the following functions: uptake and processing of antigens, migration, and subsequent presentation of these antigens to naïve CD4^+^ T lymphocytes. This interaction activates the lymphocytes and induces their differentiation into type 2 cells, consequently disrupting the balance between type 1 and type 2 cells (11). This imbalance between type 1 and type 2 cells is considered a critical immunological factor in the etiology of asthma.

An imbalance between type 1 and type 2 immune responses is characterized by diminished type 1 cell activity and elevated production of type 2 cytokines (12). A key step within the immune system’s regulatory processes involves type 2 cells promoting the differentiation of B cells into plasma cells, which subsequently produce antibodies. Under the influence of type 2 cytokines such as IL-13 and IL-4, plasma cells preferentially synthesize immunoglobulin E (IgE). The interaction between IgE and its high-affinity FcϵRI receptor on mast cells and basophils is pivotal in the allergic cascade. When an allergen binds to two adjacent IgE molecules, cross-linking occurs, activating mast cells and triggering the release of pre-formed bioactive mediators, including histamine, tryptases, and chymase. Furthermore, mast cells synthesize substantial amounts of inflammatory mediators such as cysteinyl leukotrienes, prostaglandin D_2_, and type 2-associated cytokines (IL-13, IL-9, IL-5, and IL-4) (13). These mediators amplify the type 2-mediated inflammatory response, leading to increased vascular permeability, excessive mucus secretion, tracheal smooth muscle contraction, and inflammatory cell infiltration. IL-4, acting as an upstream regulatory cytokine for type 2 effector cytokines, binds to its receptor and facilitates the differentiation of naïve CD4^+^ T cells into the type 2 phenotype (14). Prostaglandin D_2_, the primary prostaglandin derived from mast cells and eosinophils, shows a positive correlation with asthma severity, attack frequency, and type 2-associated inflammatory markers. IgE-mediated allergen presentation lowers the threshold for triggering allergen-specific type 2 cell responses, thereby enhancing IgE synthesis and establishing a self-perpetuating cycle central to asthma pathogenesis (15).

Additionally, an imbalance between Th17 and regulatory T cells significantly contributes to the pathophysiology of chronic obstructive pulmonary disease. The overactivation of Th17 cells enhances the inflammatory response, while a reduction in regulatory T cells weakens immune regulation, failing to inhibit inflammation effectively and contributing to the progression of the disease (16). Three innate lymphoid cell (ILC) types are also involved in asthma development. ILC1 primarily produces interferon (IFN), while ILC3 predominantly secretes IL-17 and IL-22. ILC2 releases cytokines such as IL-13, IL-4, and IL-5, which recruit and activate inflammatory cells, including mast cells and eosinophils, causing allergic airway inflammation and exacerbating asthma symptoms (2). Additionally, factors such as airway and gut microbiota composition, allergen exposure, air pollution, oxidative stress, neuroregulation, epigenetics, sex hormones, and age are closely related to asthma (17).

## 3 TLRs

TLRs were first discovered in fruit flies in 1988, with the discovery of TLR4 in humans in 1997 (18, 19). To date, researchers have revealed the existence of 10 functionally active TLRs (TLR1 through TLR10) in humans and 12 in laboratory mice (20). TLR1, TLR2, TLR4, TLR5, and TLR6 are predominantly expressed on the cell surface of various immune cells, where they primarily recognize lipids and protein constituents, whereas TLR3, TLR7, TLR8, and TLR9 are expressed within endosomes and activated by specific nucleic acid ligands, including viral double-stranded RNA, dsDNA, sense single-stranded RNA, and bacterial unmethylated cytosine-phosphate-guanine (CpG) DNA respectively (21–25).

TLRs function as pattern recognition receptors that identify PAMPs and DAMPs, thereby initiating immune signaling and promoting the maturation and activation of immune cells. Upon recognizing PAMPs or DAMPs, TLRs activate intracellular signaling cascades by inducing structural alterations in their Toll/IL-1 receptor (TIR) domain, which enables the recruitment of cytoplasmic adapter proteins (26). The TLR signaling pathway is classified into two distinct categories based on the associated adaptor proteins: the myeloid differentiation factor 88 (MyD88)-dependent pathway, present in all TLRs with the exception of TLR3, and the MyD88-independent pathway, referred to as the TIR-domain-containing adapter-inducing IFN-β (TRIF)-dependent pathway (27). The MyD88-dependent pathway activates mitogen-activated protein kinase (MAPK) and nuclear factor-κB (NF-κB), leading to the production of proinflammatory cytokines. The TRIF-dependent pathway stimulates the synthesis of type I IFNs and inflammatory cytokines mediated by IFN regulatory factor 3 (IRF3) (28). Under pathological conditions, TLRs contribute to allergic reactions, inflammatory responses, and autoimmune diseases by identifying microbial components or endogenous molecules (29).

## 4 Role of TLRs in asthma

Multiple studies have demonstrated a correlation between TLRs and asthma as well as chronic respiratory inflammation, with genetic polymorphisms in TLRs influencing susceptibility to and severity of asthma. These variations complicate the effective management of asthma-related respiratory inflammation (30, 31). Increasing evidence indicates that TLR family members and their associated signaling pathways perform pivotal functions in the context of asthma pathobiology.

### 4.1 TLRs

#### 4.1.1 TLR1

TLR1 is expressed in myeloid cells, T and B cells, natural killer cells, and microglia and astrocytes and is involved in the recognition of cell wall constituents, including viral proteins and bacterial lipoproteins (32–34). The expression of TLR1 on peripheral blood mononuclear cells is significantly higher in patients with asthma than that in healthy individuals, indicating that TLR1 plays a role in the pathogenesis of asthma (35). However, *TLR1/2* mRNA treatment has demonstrated protective effects on asthma induced by house dust mites in mice (36).

Furthermore, the polymorphism of the *TLR1* gene is associated with the risk of asthma. Kormann et al. confirmed that the *TLR1* single-nucleotide polymorphisms (SNPs), such as rs5743594, rs5743595, and rs4833095, have a protective effect on atopic asthma (37), but the variant *TLR1* rs5743618 may increase the risk of asthma at the age of 11–13 years after infant bronchiolitis (38, 39). The aforementioned studies indicate that, on the one hand, TLR1 recognizes pathogens and is highly expressed in patients with asthma, suggesting its potential role in promoting inflammation or in the pathogenesis of asthma, but on the other hand, the TLR1/2 signaling pathway has been confirmed to have protective effects on asthma, and *TLR1*-specific gene polymorphisms are associated with either protection against asthma or risk of asthma. These seemingly contradictory findings suggest that the specific role of TLR1 in asthma may be regulated by multiple factors, and it is worthy of further in-depth exploration.

#### 4.1.2 TLR2

TLR2 is broadly expressed on the surface of diverse cell types, including both immune cells, such as myeloid cells and T cells (40, 41) and non-immune cells, such as epithelial, endothelial, and nerve cells (42). Typically, TLR2 forms functional heterodimers with TLR1 or TLR6 on the cell surface. This heterodimerization not only broadens the spectrum of PAMPs recognized by TLR2 but also diversifies the resulting downstream signaling cascades (43). This widespread distribution underscores the significant role of TLR2 in linking innate immunity, tissue homeostasis, and disease pathogenesis. TLR2 transduces signals from various molecules, including lipoproteins and cell wall components of Gram-positive bacteria, peptidoglycans and lipoarabinomannan from mycobacteria (44, 45), and the high mobility group protein B1 (HMGB1) (46).

In the context of asthma, the role of TLR2 is particularly evident. Pauline et al. demonstrated that specific recognition of *Aspergillus fumigatus* conidia PAMPs by the TLR2/MyD88 pathway elevates IL-10 levels while reducing lung eosinophilia and type 2 responses (47). Furthermore, microRNA-146a (miR-146a), a key regulatory factor in ovalbumin (OVA)-induced allergic asthma models, alleviates asthma symptoms by modulating the TLR2 signaling pathway (48). Genetic studies reveal that TLR2 deficiency enhances IgE production but suppresses IgG1 class switching following OVA sensitization (49). Specific polymorphisms, such as *TLR2* rs3804100, are associated with allergic asthma (50), whereas *TLR2* rs4696480 shows a significant link to asthma susceptibility (51). Interestingly, genetic variants within the TLR2-associated heterodimer network (including TLR1 and TLR6) confer strong protection against atopic asthma (37).

Collectively, these studies suggest that TLR2 predominantly exerts a protective role in asthma. Its activation can mitigate allergic inflammation through multiple mechanisms, including inhibiting type 2 responses, regulating antibody class switching, and inducing anti-inflammatory factors such as IL-10. However, the precise impact of TLR2 activation likely depends on factors such as the timing of microbial exposure and individual genetic background.

IgE and its high-affinity Fc receptor, FcϵRI, are pivotal in asthma pathogenesis. Genome-wide association studies have shown that the Fcϵ receptor Ia (*FCER1A*) gene, encoding the ligand-binding subunit of the high-affinity IgE receptor, is the main susceptibility locus for serum IgE levels. Genetic polymorphisms of the *FCER1A* gene may affect IgE levels related to asthma (52). In atopic dermatitis, an interaction between the *TLR2* rs4696480 locus and the *FCER1A* rs2252226 locus has been linked to disease severity (53). Concurrently, TLR2 activation induces FcϵRI downregulation in human Langerhans cells (54). A suggested mechanism posits that TLR2 regulates the type 1 and type 2 balance. Nevertheless, this is complicated by the fact that TLR2 drives a type 1 deviation during the chronic phase of atopic dermatitis (55), contrasting with generally type 2-polarized immune response of asthma (56). TLR2 modulates FcϵRI expression in airway antigen-presenting cells. If TLR2 downregulates FcϵRI, it could attenuate IgE-mediated mast cell activation, thereby mitigating acute allergic responses. The relevance of the *TLR2* rs4696480 polymorphism established in atopic dermatitis (57) raises the possibility of a similar effect in asthma, potentially influencing IgE levels via FCER1A expression regulation. However, further experimentation is required to confirm the direct regulatory effect of TLR2 on FcϵRI expression in the context of asthma.

#### 4.1.3 TLR3

TLR3 is located on the endosomal membranes of epithelial cells, neuroglial cells, neurons, and dendritic cells and can recognize viral double-stranded RNA, polyinosinic:polycytidylic acid. This receptor plays a crucial role in autoimmune diseases, viral infections, and the development of asthma (58–61). Abnormal activation of the TLR3 signaling pathway and excessive activation of its downstream signaling factor TRIF can trigger the recruitment of local inflammatory cells and excessive synthesis of proinflammatory mediators, thereby causing various inflammatory diseases including asthma (62). In animal models of asthma, stimulation of the TLR3/TRIF signaling pathway has been demonstrated to exacerbate airway inflammation, promote inflammatory cell infiltration, and worsen the severity of the asthma response (62). After TLR3 is activated, significant infiltration of inflammatory cells occurs in the lung tissue, which subsequently leads to epithelial damage and histamine release. Excessively released inflammatory mediators promote the migration and differentiation of lung fibroblasts and airway matrix cells, enhance extracellular matrix synthesis, and cause thickening and fibrosis of the airway wall, thereby further exacerbating the severity of asthma (62, 63). Sugiura et al. also confirmed that in asthma, activation of the TLR3/TRIF signaling pathway could affect airway remodeling by promoting the differentiation of myofibroblasts (64).

Furthermore, studies have also revealed the important role of the TLR3/NF-κB/IRF3 signaling pathway in the progression of viral-induced asthma (65, 66). For example, respiratory syncytial virus infection could upregulate the expression of TLR3, resulting in the overexpression and release of downstream inflammatory factors through the TLR3/NF-κB/IRF3 pathway in asthmatic mice; this process also enhances extracellular matrix synthesis, further aggravating asthma symptoms (67). Additionally, Ramu et al. found that the TLR3/TAK1 signaling pathway regulates the production of IL-33 in bronchial smooth muscle cells during nasal rhinovirus infection (68).

The contribution of TLR3 genetic variants to asthma pathogenesis remains an area of investigation, with evidence showing some heterogeneity. While one study in Chinese Han patients failed to detect a significant association between TLR3 SNPs and asthma susceptibility or severity (69), other investigations have reported positive links. Specifically, research on aspirin-tolerant asthma found that TLR3 polymorphisms, particularly -299698G>T and 293391G>A [Leu412Phe] were associated with various respiratory phenotypes encompassing asthma (70). Moreover, both Canadian family-based analyses and an Australian population-based case-control study associated the rs1519309 SNP within TLR3 with atopic asthma (71). Functionally, TLR3 is known to mediate viral recognition and subsequent inflammatory responses, processes that are believed to significantly influence asthma progression. Indeed, excessive activation of TLR3 has been shown to exacerbate inflammation and fibrosis. However, despite correlations observed between TLR3 genetic variants and various asthma and allergic phenotypes, the specific impact of these variants on asthma susceptibility and severity exhibits considerable heterogeneity across different populations and studies. This inconsistency underscores the complex and multifactorial nature of TLR3’s regulation within the context of asthma pathogenesis.

#### 4.1.4 TLR4

TLR4 is primarily expressed on myeloid-derived immune cells, including macrophages, monocytes, and dendritic cells (72–74) and also present on non-immune cells such as microglia, astrocytes, neurons, and endothelial cells (75). The outer membrane component of Gram-negative bacteria, lipopolysaccharide (LPS), serves as a specific ligand for TLR4 (76). Upon LPS binding, TLR4 activates the MyD88-dependent signaling pathway, triggering a cascade that activates downstream kinases. This cascade facilitates the nuclear translocation of NF-κB-associated factors, enabling their regulation of proinflammatory gene expression, particularly for *IL-6*, *IL-1β*, and tumor necrosis factor-α genes (77). TLR4 triggers immune responses via two distinct signaling pathways: the TRIF-dependent and the MyD88-dependent pathways (78). Stimulation of the MyD88/NF-κB pathway enhances the production of proinflammatory cytokines (77, 79), whereas the TRIF pathway contributes to asthma exacerbation (80).

Research by McAlees JW et al. highlights the differential roles of TLR4 depending on the cell type involved. Their findings indicate that TLR4 expression in hematopoietic cells is crucial for neutrophilic airway inflammation, observed both following LPS exposure and in Th17-driven neutrophilic responses to house dust mite lysates and OVA. In contrast, TLR4 expression by airway epithelial cells plays a key role specifically in robust eosinophilic airway inflammation when these same allergens are used for sensitization and challenge (81). However, broader research indicates that TLR4 activation predominantly promotes eosinophilic responses and type 2 inflammation. Specifically, studies demonstrate that LPS binding to TLR4 promotes type 2 immune responses, thereby exacerbating allergic respiratory inflammation (79). The cytokines and chemokines produced during type 2 responses are pivotal in asthma development, facilitating eosinophil recruitment and survival, promoting antigen-specific antibody production, and inducing mucus cell proliferation in the bronchial epithelium (82). Beyond recognizing exogenous microbial PAMPs, TLR4 also detects endogenous DAMPs (83), including HMGB1 and cellular heat shock proteins (84). The interaction between TLR4 and HMGB1 activates the NF-κB pathway, leading to the synthesis of inflammatory mediators and exacerbation of asthma symptoms. Furthermore, suppression of HSF1 has been shown to worsen OVA-induced airway inflammation and hyperresponsiveness in mice, likely through HMGB1 upregulation and activation of the TLR4/MyD88/NF-κB pathway (85). Aberrant activation of the TLR2/TLR4 signaling pathway can result in the excessive production of inflammatory factors, such as IL-1β, IL-5, IL-8, IL-4, and IL-13, and chemotactic mediators by neutrophils, monocytes, and type 2 lymphocytes within the airway. These mediators recruit neutrophils, stimulate goblet cell hyperplasia, and amplify the type 2 immune response. Consequently, this cascade leads to the synthesis of allergen-specific IgE and IgG1, increasing airway hyperresponsiveness and potentially triggering acute asthma attacks (77, 86). Collectively, these studies demonstrate that TLR4, by bridging innate and adaptive immunity and responding to diverse stimuli (both PAMPs and DAMPs), plays a multifaceted role in asthma pathophysiology, influencing inflammation, immune deviation, inflammatory cell recruitment, airway remodeling, and hyperresponsiveness, establishing TLR4 as a critical regulatory node.

Additionally, polymorphisms in the *TLR4* gene are significantly correlated with an elevated risk of asthma (87). *TLR4* rs4986791 was found to be significantly associated with asthma susceptibility in a meta-analysis, especially in the Asian population (88). The findings from a comparative analysis demonstrate an association between the *TLR4* rs4986791 variant and bronchial asthma risk among Chinese children (89). *TLR4* rs1927911 is also associated with antibiotic exposure and bronchiolitis in childhood asthma (90). Although mice expressing cosegregating SNPs D298G and T399I in *TLR4* have enhanced responses to the house dust mite allergen, their responses to OVA and LPS were not significant (91). These findings indicate that *TLR4* polymorphic variants exhibit differential interactions with allergic stimuli, potentially modulating immune responses in a genotype-dependent manner. Although existing studies (including population association analyses and animal models) have provided strong evidence for the role of TLR4 in asthma, there are still some limitations. For instance, population studies are mostly correlational analyses, making it difficult to determine causal relationships.

#### 4.1.5 TLR5

TLR5 is a receptor that recognizes bacterial flagellar proteins and is not only expressed on the surface of myeloid cells, but also on the surface of epithelial cells, microglia, and astrocytes. It is abundantly expressed in airway epithelial cells and weakly expressed in alveolar macrophages (92). Shikhagaie et al. discovered that TLR5 is widely expressed in the lungs, but its expression is reduced in severe asthma. In patients with severe asthma, a significantly negative correlation is present between intracellular TLR5 immunoreactivity and IgE (93). This might be due to the fact that patients with severe asthma have insufficient TLR signaling during viral or bacterial infections, resulting in a reduced anti-allergic type1 response and a weakened or impaired immune defense mechanism. The binding of the flagellin protein to its receptor TLR5 affects the transcriptional profile of human primary epithelial cells, including genes encoding chemokines, matrix metalloproteinases, and antimicrobial molecules, thus inducing the expression of proinflammatory cytokines and chemokine mRNAs and promoting the secretion of granulocyte-macrophage colony-stimulating factor, C-X-C motif chemokine ligand-5, C-C motif chemokine-5, and C-X-C motif chemokine ligand-10, which may contribute to airway inflammation and remodeling (94). Bacterial flagellin in the household environment initiates allergic responses to indoor allergens in a TLR5-dependent manner, thereby promoting the development of allergic asthma (92). In OVA-specific asthmatic mouse models, the activation of TLR5 by bacterial products (flagellin) exacerbates allergic airway inflammation (95).

While the recognition of flagellin generally of TLR5 promotes inflammation in asthma, genetic variations in the TLR5 gene reveal a more complex picture, sometimes offering protection. For instance, a dominant-negative polymorphism (rs5744168) is linked to alleviated asthma symptoms in patients (95). Interestingly, another TLR5 variant (rs5744174) might increase susceptibility to bronchiolitis not caused by respiratory syncytial virus (non-RSV bronchiolitis), but this variant does not appear to be associated with the subsequent development of asthma following such bronchiolitis (96).

#### 4.1.6 TLR6

TLR6 is mostly present on the cell membrane of myeloid cells and also expressed on epithelial cells, microglia, and astrocytes, where it forms heterodimers with other TLRs, particularly TLR2 (30). TLR6 forms heterodimers with TLR2 to recognize specific pathogen-associated components such as diacyl lipopeptides, lipoteichoic acid, and zymosan. Chun et al. examined the expression of TLR6 on CD14^high^ cells in patients with asthma and found that TLR6 expression in the asthma group was significantly lower than that in the control group, and the expression level of TLR6 was statistically significant among patients with mild, moderate, and severe asthma (35). In the asthma model of TLR6^-/-^ mice induced by fungal or house dust mite antigens, airway hyperresponsiveness, inflammation and remodeling worsened significantly, but the levels of IL-23 and IL-17 in the whole lung decreased significantly. Exogenous IL-23 treatment restored the production of IL-17A in asthma TLR6^-/-^ mice, significantly reducing airway hyperresponsiveness, inflammation and pulmonary fungal burden (97). This indicates that TLR6 may play a certain protective or regulatory role in asthma through the IL-23/IL-17 pathway, and its low expression may be associated with the aggravation of asthma.

In the development of asthma, TLR6 not only regulates the immune response through signaling pathways, but its genetic polymorphisms also jointly influence the susceptibility to the disease along with environmental exposure. In children exposed to a farm environment, those carrying the *TLR6* rs1039559 T-allele and the *TLR6* rs5743810 C-allele have a lower risk of early-onset asthma compared with healthy children (98). The polymorphism of *TLR6* rs5743810 has some exploratory correlation with airway reactivity (99) and a weak correlation with childhood asthma (100). Perhaps this conclusion (such as reducing risks) requires a larger sample size and more rigorous environmental control for verification.

#### 4.1.7 TLR7/TLR8

TLR7 is mostly expressed in human plasmacytoid dendritic cells and, to a lesser degree, T cells, B cells, neutrophils, eosinophils, and mononuclear macrophages (101). TLR8 is predominantly expressed in myeloid dendritic cells, neutrophils, and monocytes (102). TLR7 and TLR8 recognize single-stranded RNA as their natural ligand (24). Yan et al. employed a combined analysis approach based on the gene expression profiles of induced sputum derived from the comprehensive Gene Expression Omnibus databases (GSE76262 and GSE137268) and found that reduced TLR7 expression correlated with airway eosinophilic inflammation and lung function in asthma (103). In a mouse asthma model induced by dust mites, TLR7 expression was significantly downregulated. TLR7-deficient asthmatic mice exhibited substantial inflammatory cell infiltration in the lungs, accompanied by elevated levels of IL-4, IL-10, IL-12, and IFN-γ, as well as increased phosphorylation of inhibitory κB kinase-α and NF-κBp65 expression. Importantly, the administration of a TLR7 agonist reversed these detrimental effects, suggesting that TLR7 upregulation mitigates asthma inflammation by inhibiting the NF-κB signaling pathway (104). Consistent with this finding, another study demonstrated that the interaction between TLR7 and its ligand alleviates eosinophilia and allergen-induced airway hyperreactivity in asthma, a process mediated by MyD88-dependent but MK2-independent signaling pathways (105). However, the interaction of TLR8 with its ligand promotes neutrophil proliferation, induces the secretion of chemokines and neutrophil elastase, triggering an airway inflammatory response that may counteract the protective effect of TLR7 (106). In rhinovirus-induced asthma, the IFN response is defective. Administration of the TLR7/8 agonist R848 to peripheral blood mononuclear cells from preschool asthmatic children was found to decrease *IFN-αR* mRNA levels while inducing *IFN-λR1* mRNA. These findings suggest that targeting TLR7/8-mediated modulation of type I/III IFN signaling pathways could represent a novel therapeutic strategy to enhance antiviral immunity in pediatric asthma (107).

Although the available evidence suggests a protective role of TLR7 and a proinflammatory effect of TLR8, several key issues require further exploration. Firstly, the expression and function of TLR7/8 are likely influenced by factors including cell type, the underlying inflammatory milieu (e.g., eosinophilic versus neutrophilic predominance), and disease stage. Furthermore, the observed correlations in the literature—such as the association between TLR7 expression and eosinophilic inflammation—are not yet established as causal relationships. Secondly, although evidence supports the proinflammatory properties of TLR8, whether it directly counteracts the protective effects of TLR7 within the human body, and if this interaction is truly and universally significant, requires further direct investigation.

This complexity is further highlighted by genetic association studies. For example, a study found that an increased frequency of *TLR7* gene polymorphisms is correlated with a higher risk of asthma in preschool children following infant bronchiolitis (108, 109). Conversely, although case-control and case-only studies found no significant association between *TLR7* and *TLR8* genetic variations and overall asthma susceptibility, these same variations were linked to asthma-related phenotypes. Specifically, polymorphisms in both *TLR7* and *TLR8* were associated with eosinophil count, serum IgE levels, lung function, and asthma severity (110). This suggests that genetic variations in *TLR7* and *TLR8* may indeed contribute to asthma pathogenesis, particularly influencing disease characteristics rather than initial susceptibility.

#### 4.1.8 TLR9

TLR9 is mainly found in monocytes, plasmacytoid dendritic cells, B cells, microglia, astrocytes, and neurons. It recognizes self-DNA within immune complexes and unmethylated CpG DNA from viral, bacterial, and parasitic sources (100). When TLR9 binds to unmethylated CpG DNA on the surface of plasmacytoid dendritic cells and B cells, it can trigger type 1 immune responses and promote the differentiation and development of regulatory T cells. This process is accompanied by the production of IFN-γ and the release of IL-10 and transforming growth factor-β, and these cytokines work together to inhibit type 2 cell responses and alleviate asthma symptoms (111). Consistent with this anti-inflammatory potential, intranasal administration of CpG can alleviate experimental fungal asthma in a TLR9-dependent manner (112).

However, the role of TLR9 in asthma is complex. Some studies suggest that the TLR9–IL-2 axis may contribute to the deterioration of allergic asthma by exacerbating excessive IL-17A production (113). Furthermore, evidence indicates that TLR9 may regulate allergic airway inflammation by activating the NLRP3 inflammasome and inducing oxidative stress (114). Despite these complexities, TLR9 agonists hold promise as potential immunomodulators or vaccine adjuvants, exhibiting promising prospects in the research of immunotherapy for allergic diseases (115).

Additionally, genetic factors play a role, as specific *TLR9* polymorphisms may influence the clinical manifestations of childhood asthma. For instance, the *TLR9* rs187084 polymorphism is associated with the control of childhood bronchial asthma to some extent (116), and the overall *TLR9* SNP profile is significantly correlated with susceptibility to childhood asthma (109). This genetic link is further supported by studies on bronchiolitis-induced wheezing, which suggest that the *TLR9* rs187084 gene polymorphism may be a potential genetic factor in its occurrence and development (117).

#### 4.1.9 TLR10

TLR10 is a recently identified member of the human TLR family. Currently, it is regarded as the only orphan receptor in this family, and its exact function and natural ligands remain unclear (118). TLR10 is expressed as a type I transmembrane protein in various immune cells such as B cells and dendritic cells (119). Its mRNA is highly expressed in lymphoid tissues such as the spleen, lymph nodes, thymus, and tonsils.

Although there is a lack of in-depth analysis of its biological function, genetic studies have found that the polymorphisms of the human *TLR10* gene are associated with various disease states, including bacterial infections, asthma, autoimmune diseases, and cancer (120–122). Lazarus et al. confirmed that *TLR10* gene variations are related to asthma susceptibility (122). In particular, TLR10 is associated with the occurrence of bronchiolitis post-asthma, which may increase the risk of developing asthma in infants who have suffered from bronchiolitis before the age of 11–13 (38, 108, 116). These findings suggest that TLR10 may play a potential role in the pathogenesis of respiratory diseases, especially in the onset of childhood asthma, and warrant further in-depth research.

### 4.2 TLR signaling pathways involved in asthma

#### 4.2.1 MyD88 pathway

The TLR signaling pathway typically initiates downstream signal transduction through two major adaptor proteins: MyD88 and TRIF. Among these, MyD88 plays a central role in signal transduction and is essential for the activation of most TLRs (123). Structurally, MyD88 contains three distinct functional domains: an N-terminal death domain, a central intermediate region, and a C-terminal Toll/IL-1 receptor (TIR) domain. The cytoplasmic region of TLRs shares significant homology with the TIR domain of the IL-1 receptor (IL-1R) family, which is critical for downstream signaling. TIR domain-containing adaptor proteins are essential for TIR-mediated signaling, as they are required for the activation of TLR4 and TLR2 pathways, but not for TLR5, TLR7, or TLR9 (124, 125).

Most TLRs, including TLR1/2, TLR4, TLR5, TLR6/2, TLR7/8, and TLR9, transmit signals through the MyD88-dependent pathway (126). This pathway activates the NF-κB and MAPK signaling cascades, leading to the production of pro-inflammatory cytokines such as IL-1β, IL-6, and TNF-α (47, 86, 104, 115, 127). In the context of asthma, dysregulation or aberrant activation of the MyD88-dependent signaling pathway has been implicated in the development of immune imbalance, characterized by enhanced type 2 immune responses, suppression of regulatory T cell function, and exacerbated airway inflammation and remodeling. Specifically, TLR4, which recognizes both exogenous ligands such as LPS from Gram-negative bacteria and endogenous danger signals like HMGB1, activates the NF-κB and MAPK pathways via the MyD88-dependent pathway. This activation promotes Th2 cell differentiation and IgE production, which are key features of allergic asthma (85, 86). Similarly, TLR2/1 and TLR2/6, which recognize bacterial lipoproteins and peptidoglycans, also signal through the MyD88 pathway, inducing the expression of IL-12 and IFN-γ. Under certain conditions, this may suppress type 2 responses, but excessive activation can lead to chronic inflammation (47, 49). Furthermore, TLR7/8, which detects single-stranded RNA from viruses, activates the MyD88 and IRF7 pathways, resulting in the production of IFN-α/β and IL-12. This signaling is particularly relevant in viral-induced asthma, highlighting the critical role of MyD88-dependent pathways in linking microbial exposure to immune dysregulation in allergic diseases (105, 107).

#### 4.2.2 TRIF pathway

TRIF is extensively expressed across various cell types and is located within the cytoplasm under resting conditions. The TRIF signaling pathway is shared between TLR3 and TLR4 (128). Specifically, TRIF can directly interact with TLR3 or associate with TLR4 indirectly through the TRIF-associated adaptor molecules (6). Upon binding to TLR4, activation of the TRIF pathway occurs within the endosomal compartment following TLR4/MD2 complex internalization. Activation of the TRIF pathway involves activation of tumor necrosis factor receptor-associated factor 3, induction of nuclear translocation of IRF3, and recruitment of IFN-β. These processes are mediated by TRIF-associated adaptor molecules and TIR domain-containing adaptor proteins (129). The TRIF-dependent pathway can activate IRF3 and NF-κB, inducing proinflammatory cytokine gene expression and type I IFN production. This mechanism exacerbates asthma (130).

Although the role of TLRs in asthma has been extensively investigated, several controversies remain unresolved, highlighting the complexity of TLR-mediated immune regulation in allergic diseases. First, some TLRs exhibits a dual and context-dependent role in asthma: while it can drive pro-inflammatory responses by activating NF-κB and MAPK pathways, it may also exert protective effects (95, 96). This duality underscores the need for a more nuanced understanding of functional outcomes of TLRs in different disease contexts. Second, cross-regulation among TLR signaling pathways adds another layer of complexity. TLRs can interact in both synergistic and antagonistic manners, leading to divergent immune responses. Such interactions may contribute to the heterogeneity of asthma phenotypes and complicate the interpretation of experimental findings (105, 106). Finally, individual variability and epigenetic regulation further complicate the picture. The activity of TLR signaling pathways may be influenced by epigenetic modifications which may alter gene expression and immune cell function. These factors not only contribute to inter-individual differences in disease susceptibility but also highlight the importance of integrating epigenetic perspectives in future TLR research.

### 4.3 The association of TLRs with microbial exposure and epigenetic regulation in allergic diseases

TLRs are pivotal in shaping immune responses within allergic diseases, acting as sentinels that recognize microbial components present in the environment. Consistent with the hygiene hypothesis, diminished early-life microbial exposure is thought to skew immune development towards type 2 imbalance, thereby increasing susceptibility to allergic conditions like asthma (131). The TLR signaling pathway directly influences the trajectory of the immune response upon encountering microbial molecules. For example, Fuchs et al. demonstrated that TLR2/6 agonists could suppress type 2 inflammation, concurrently reducing airway hyperresponsiveness and mucus secretion (132). This interplay further underscores the complexity, as the combined application of TLR agonists with miR-146a mimics was found to attenuate OVA-induced asthma inflammation in mice (48).

Crucially, the relationship between TLRs, microbial exposure, and immune outcomes is deeply intertwined with epigenetic regulation. Maternal smoking, for instance, can induce aberrant TLR signaling in newborns, a key innate mechanism for microbial recognition, potentially laying the molecular groundwork for heightened infant infection susceptibility (133). Conversely, prenatal exposure to non-pathogenic microorganisms, engaging TLR-dependent pathways, significantly lowers the asthma risk of offspring (134). This protective effect of microbe-TLR interaction appears intrinsically linked to epigenetic modifications.

The expression of miR-146a, a common regulator of both TLR4 and ST2 signaling, serves as a prime example. Its levels are modulated by the intensity of microbial encounters early in life, suggesting it may be a critical epigenetic link connecting early environmental stimuli to adult asthma susceptibility (135). Furthermore, when air allergens and respiratory viruses activate TLRs, the downstream oxidative stress can induce modifications like the oxidation of guanine bases within inflammatory gene regulatory regions. These modifications act as epigenetic marks associated with inflammation induction. Targeting the pathways that induce this epigenetic reprogramming offers a potential therapeutic strategy to reverse airway remodeling seen in various chronic airway diseases (136).

In essence, TLRs function as environmental sensing hubs, orchestrating the reprogramming of epigenetic markers, such as miR-146a, in response to microbial exposure. This dynamic regulation significantly influences the initiation and progression of asthma. However, the intricate details of these mechanisms undoubtedly warrant further investigation.

## 5 TLR-related asthma treatment strategies

### 5.1 TLR antagonists/inhibitors

The involvement of TLRs in the pathogenesis of asthma is well-documented, with excessive activation of TLRs being closely associated with the development of the disease. Therefore, blocking TLR signaling pathways has emerged as a potential therapeutic strategy (137).

Among various TLRs, TLR4 has been the most extensively studied in the context of asthma treatment. Several compounds have been investigated for their ability to modulate TLR4 signaling and alleviate asthma symptoms. For instance, saxagliptin, a dipeptidyl peptidase-4 inhibitor, has been shown to significantly reduce airway inflammation in OVA-induced asthmatic mice by inhibiting the TLR4/reactive oxygen species/NF-κB signaling pathway. It also mitigates oxidative stress in lung tissue and lowers the levels of NF-κB and TLR4, suggesting its potential as a therapeutic agent (138). Resveratrol, a natural polyphenol found in grape skins, berries, and nuts, has demonstrated therapeutic potential in various pulmonary diseases, including pulmonary fibrosis, atherosclerosis, and pulmonary hypertension (139–141). In the context of asthma, high-dose resveratrol can suppress the production of inflammatory cytokines by inhibiting the HMGB1/TLR4/NF-κB pathway, thereby reducing airway inflammation and remodeling (142). Paeonol, an active compound derived from Paeonia suffruticosa, has been shown to downregulate TLR4 expression and block the nuclear translocation of NF-κB in asthma models. It also reduces the phosphorylation of p38 and extracellular signal-regulated kinase (ERK), leading to improved symptoms in OVA-induced asthma (143).

In addition to small-molecule inhibitors, other therapeutic approaches targeting TLR signaling include the use of probiotics and prebiotics, which have been shown to suppress inflammatory cell infiltration and reduce the production of inflammatory mediators, suggesting potential benefits for asthma patients (77). Small-molecule compounds that modulate TLR activity, particularly those targeting TLR7, TLR8, and TLR9, are also being explored for the management of inflammation-related diseases, including asthma (6, 144).

### 5.2 TLR agonists

Currently, several studies are exploring the therapeutic potential of TLR agonists in the treatment of asthma. Among them, the TLR2 agonist Pam3CSK4 has shown promising effects. Animal studies by Liao et al. demonstrated that Pam3CSK4 reduces type 2 inflammatory cytokines and Th17 cell levels, alleviates airway inflammation, and decreases airway hyperresponsiveness, suggesting its potential in asthma management (145). In addition, TLR4 agonists are primarily used as adjuvants in allergic vaccines to promote immune tolerance to TLR4 ligands and suppress TLR4 receptor expression. Monophosphoryl lipid A, a TLR4 agonist, has shown significant therapeutic effects when used as an allergen adjuvant, particularly in patients with asthma who exhibit elevated TLR4 expression in the airways. Resiquimod, a dual agonist of TLR7 and TLR8, has also been investigated for its anti-inflammatory effects in experimental asthma models. It has been shown to reduce Th2 inflammatory cytokine release, decrease eosinophil infiltration in the bronchus, and lower serum IgE levels (146). Despite these positive findings, the use of TLR agonists in asthma treatment remains controversial due to potential adverse effects. For example, resiquimod may cause local irritation, such as erythema and itching, when applied topically. In summary, while TLR agonists show promise as potential therapeutic agents for asthma, further research is needed to fully evaluate their safety, efficacy, and optimal application in clinical settings.

### 5.3 Mucin 1

Mucin 1 is a transmembrane glycoprotein of the mucin family, composed of an N-terminal extracellular subunit and a C-terminal cytoplasmic subunit (147). It is expressed not only in epithelial cells but also in immune cells such as dendritic cells and macrophages (148). Mucin 1 exerts anti-inflammatory effects by inhibiting TLR activation during bacterial and viral infections (149). Importantly, it plays a key role in asthma pathogenesis by regulating the TLR4/MyD88/NF-κB signaling pathway, thereby suppressing neutrophil-mediated airway inflammation. These findings suggest that Mucin 1 may represent a promising therapeutic target for asthma treatment.

### 5.4 HMGB1

HMGB1 is a highly conserved nuclear protein that can be actively secreted by macrophages, monocytes, and other immune cells. It is released passively from damaged necrotic tissue cells (150, 151). As an endogenous DAMP, HMGB1 contributes to the progression of allergic respiratory disorders by interacting with TLR4 and the receptor for advanced glycation end-products. The activated TLR4/MyD88/NF-κB signaling pathway is crucial in HSF1/HMGB1-mediated asthma (85). Inhibition of the TLR4/MYD88/NF-κB pathway has demonstrated efficacy in alleviating neutrophil-induced airway inflammation in individuals with asthma (147). Experimental animal studies show that HMGB1 interacts with advanced glycation end-products, TLR2, and TLR4, leading to the activation and production of proinflammatory factors (152). Therefore, inhibiting HMGB1 can mitigate OVA-induced airway inflammation and hyper-responsiveness (84). Anti-HMGB1 antibodies not only inhibit the activation of HMGB1-mediated pathways but also reduce type 2 cytokine accumulation, inflammatory cell infiltration, and mucin production in diisononyl phthalates-induced asthma models, thereby alleviating airway hyperresponsiveness and lung tissue damage (153).

### 5.5 miRNAs

microRNAs (miRNAs), approximately 22 nucleotides long, are noncoding RNA molecules that regulate gene expression at the post-transcriptional level. They primarily bind to the 3′-untranslated region of target mRNAs, causing their translational inhibition or degradation (154). To date, most miRNAs have been identified within cells, exerting a considerable influence on asthma prognosis, treatment, and therapeutic drug development (155). For example, miR-21, miR-223, and let-7a have been implicated in the development and pathogenesis of various asthma forms (156). MiRNAs may contribute to alleviating asthma-related inflammation by modulating airway epithelial cells and regulating key signaling molecules, including forkhead box protein C1, PI3K, AKT, NF-κB, cyclin D1, and transforming growth factor β1. Additionally, TLR4 has been recognized as a direct target for miR-20a and miR-217.

### 5.6 Nanodevices

Asthma is a persistent inflammatory disorder affecting the respiratory tract, traditionally managed with inhaled corticosteroids and bronchodilators. However, these treatments have limitations, such as systemic side effects, brief retention time in the pulmonary system, and poor patient compliance. Based on the distinctive anatomical features of the lungs (encompassing a vast alveolar surface area, thin epithelial barrier, and low proteolytic activity (157), nanoparticles present a promising strategy for asthma treatment through targeted drug delivery and functional design. Among organic nanoparticles, liposomes and polymer-based nanoparticles have exhibited significant potential by encapsulating glucocorticoids (GCs) or immunomodulators. These nanoparticles enhance drug deposition rate in the airway mucosa, prolong local drug activity via controlled release mechanisms, and reduce the frequency of dosing, thereby improving treatment efficacy. Nanoparticles have emerged as a novel class of targeted and effective TLR inhibitors (158). They not only inhibit two pathways involved in TLR4 signaling (the TRIF-dependent activation of IRF and the MyD88-dependent activation of NK-κB) but also inhibit pathways associated with TLR2, TLR3, and TLR5 (6). While the application of nanoparticles in treating diseases is still in its nascent phase, their clinical efficacy requires further validation. Despite the advancements that nanotechnology has brought to the development of asthma drugs, the molecular mechanisms, performance modifications, and potential toxicological effects of nanoparticles should not be ignored.

### 5.7 GCs

GCs are currently the most effective drugs for asthma control (159), and one of its primary mechanisms involves regulating the expression of TLRs. GCs effectively inhibit airway inflammation by targeting multiple aspects of the inflammatory response, such as proinflammatory cytokine release inhibition, inflammatory cell infiltration decrease, and β2 receptor responsiveness enhancement in airway smooth muscle cells (160–162). For example, budesonide is an inhaled GC and relieves asthma by regulating the expression of TLR2, TLR4, and IL-10 in CD4^+^/CD25^+^ regulatory T cells, as well as the secretion of tumor necrosis factor-α and IL-6 from PBMCs in patients with asthma (163). GCs are commonly used to treat acute attacks of mild to moderate asthma. However, GCs have limited efficacy in many patients with severe asthma, primarily owing to the impairment of transcriptional inhibitors such as histone deacetylase 2, which results from oxidative stress-induced DNA damage (164). Additionally, GCs are associated with several side effects, including high blood pressure, central obesity, type 2 diabetes, insulin resistance, and osteoporosis. Managing these adverse effects remains a significant clinical challenge, highlighting the urgent need for alternative therapeutic strategies or the development of innovative pharmaceuticals to replace GCs.

## 6 Discussion and future prospects

**Figure 1:**
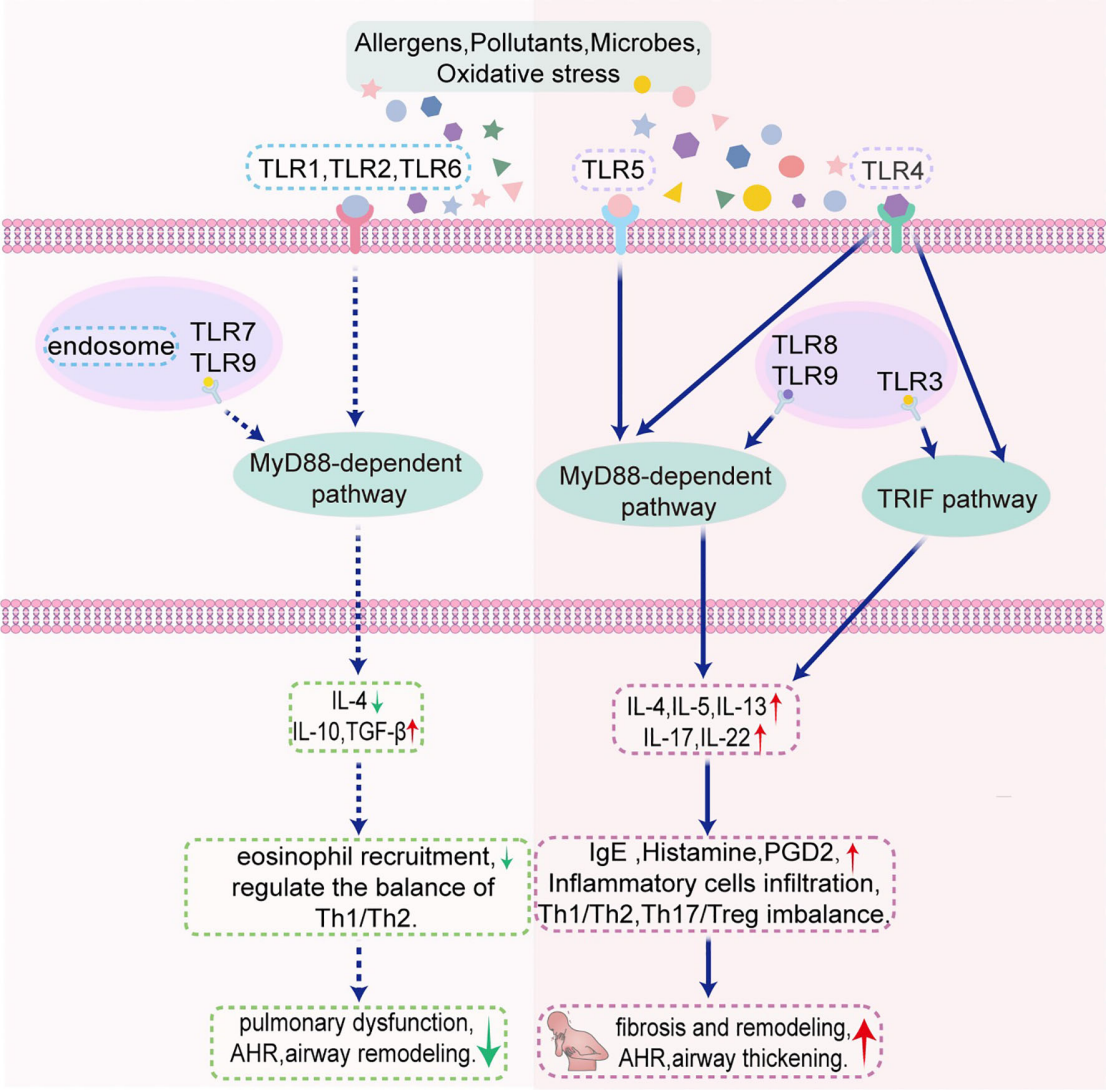
Schematic illustration of the mechanism by which TLRs regulate asthma in murine models through MyD88-dependent pathway and TRIF pathway. AHR, airway hyperresponsiveness; PGD_2_, prostaglandin D_2_; TLR, Toll-like receptor; TRIF, Toll/interleukin-1 receptor-domain-containing adapter-inducing interferon-β.

TLRs are necessary for both adaptive and innate immunity, functioning as the primary defense mechanism against microbial infections in the host. While TLRs are crucial for the amplification of immune responses and pathogen recognition, their excessive activation can destabilize the immune system and facilitate the onset of inflammatory disorders. However, most reported TLRs are implicated in asthma development. Their precise role remains debated across different studies (**Figure 1**; **Tables 1**, **2**). The controversy mainly revolves around three key aspects: whether TLR affects asthma through proinflammatory or regulatory mechanisms, the contrasting effects of different TLR subtypes on asthma progression, and the effect of environmental exposures (such as microorganisms or pollutants) on disease processes through TLR signaling (165). Several factors may contribute to these discrepancies, including variation in immune activation by different TLRs, differences in the timing and dose of microbial exposure, inconsistencies in animal models (e.g., OVA or house dust mite induction), and the presence of human TLR gene polymorphisms. While many TLR agonists and inhibitors have been developed for the management of inflammatory diseases, such as asthma, atherosclerosis, cancer, and autoimmune disorders, most studies have been conducted in animal models. However, owing to the significant differences between the animal model of asthma and human disease, further clinical translational studies are needed to validate these treatments for asthma. Additionally, environmental microbes can simultaneously activate multiple TLRs, making it more effective to develop antagonists or inhibitors that target multiple TLR signaling pathways rather than a single receptor. As a potential pharmacological target in asthma treatment, TLR modulation holds the potential to drive significant breakthroughs and expand therapeutic options for asthma management.

**Table 1:** TLRs regulate asthma through different mechanisms.

TLRs	Study model/Stimuli	Mechanism	Response	Role in disease	Ref.
TLR1	Murine HDM	Treatment with TLR1/2 mRNA	Reducing airway inflammation	Resulting in better lung function	(36)
TLR2	Murine A. fumigatus conidia	TLR2/MyD88 pathway	Upregulating IL-10 production and downregulating lung eosinophilia	Downregulating allergic lung inflammation	(47)
	Murine OVA+miR146a mimics	TLR2 pathway	Increasing Th1 cytokines and down-regulation of IL-5 and IL-13 in sorted ILC2, TLR2-related molecules being up-regulated	Relieving symptoms of asthma	(48)
	Murine OVA	TLR2-STAT3 pathway	Promotion of IgG1 and inhibition of IgE class switching	Restricting lung inflammation	(49)
TLR3	Murine OVA	TLR3/TRIF pathway	Inducing IFN-β expression and then trigger the subsequent specific immune response	Promoting the airway inflammation and remodeling in asthma	(62)
	Murine OVA+RSV	TLR3/NF-κB/IRF-3 pathway	Promoting the overexpression and release of downstream inflammatory factors, and enhance the synthesis of cell matrix	Promoting asthma attack	(67)
TLR4	Murine OVA+LPS+ probiotics	TLR4/NF-kB signaling pathway	Control AHR, eosinophil infiltration to the BALF and reduce the levels of immunoglobulins, IL-17, GTP and also decrease mucus secretion, goblet cell hyperplasia, peribronchial and perivascular inflammation and also, EPO activity	Inducing tolerance in allegro-inflammatory reactions	(77)
	Murine OVA+ propofol	TLR4/MyD88/ROS/NF-κB signaling pathway	Decreasing the number of eosinophils and the levels of IL-4, IL-5, IL-6, IL-13, and TNF-α in BALF; decreasing mucus production and goblet cell hyperplasia	Attenuating airway inflammation in a mast cell-dependent mouse model of allergic asthma	(79)
	Murine OVA+ASD	TLR4/MyD88-dependent pathway	Exacerbating lung eosinophilia	Triggering type 2 -dominant lung allergic inflammation	(82)
	Murine OVA+HSF1	HMGB1/TLR4/MyD88/NF-κB signal pathway	Reducing of IgE, inflammatory factors (IL-4, IL-5 and IL-13)	Alleviating airway inflammation and airway hyperreactivity in mice	(85)
	Murine OVA + ketamine, metformin, metformin and ketamine, triciribine, LY294002, and torin2	PI3K/AKT/mTOR and TLR4/MyD88/NF-κB signaling pathway	Reducing Penh value, total IgE, IL-4 and IL-5 levels, goblet cell hyperplasia, and mucus hyper-secretion	Attenuating asthma pathology	(86)
TLR5	Murine OVA+LPS (or FLA)	TLR5-dependent	Promoting the expression of proinflammatory cytokines and chemokines	Promoting the development of allergic asthma	(92, 95)
TLR6	Murine HDM or fungal	TLR6-dependent	The production of IL-23 and the response of Th17	Have an inhibitory effect on asthma	(97)
TLR7	Murine Dust mite antigen extract	NF-κB signaling pathway	Reducing airway inflammation and inhibiting ASMCS proliferation	Reducing airway inflammation in asthmatic mice	(104)
	Murine OVA+TLR7 ligand(S-28463)	TLR7/MYD88-dependent and TLR7/MK2-independent pathway	Decreasing airway hyperresponsiveness and eosinophilia	Reducing airway inflammation	(105)
TLR8	Peripheral blood neutrophils from asthmatic individuals LPS or different viruses	TLR-7/8-mediated	RSV triggered the release of CXCL8 and neutrophil elastase	TLR7/8 dysregulation may play a role in neutrophilic inflammation in viral-induced exacerbations	(106)
TLR9	Murine HDM	TLR9-IL-2 axis	Promoting type 2 inflammation by modulating IL-17A production	Exacerbating allergic asthma	(115)

HDM, house dust mite; OVA, ovalbumin; RSV,respiratory syncytial virus; TLR, Toll-like receptor; IRF, interferon regulatory factor; MyD88, myeloid differentiation factor 88; STAT3,transcription 3; LPS, Lipopolysaccharide; BALF, bronchoalveolar lavage fluid; ASD, Asian sand dust; HSF1, Heat shock factor 1; FLA, flagellin; HMGB1, high mobility group protein B1; IgE, immunoglobulin E; IL, interleukin; NF-κB, nuclear factor-κB; TRIF, Toll/IL-1 receptor domain-containing adapter-inducing interferon-β;ASMCS, airway smooth muscle cells.

**Table 2:** Single nucleotide polymorphisms in TLR and association with human asthma.

GENE	Polymorphism	Effect	Ref.
*TLR1*	rs5743594, rs5743595, rs4833095	Significantly associated with atopic asthma	(37)
	rs5743618	Increasing the risk of asthma at 11–13 years after infant bronchiolitis	(38, 39)
*TLR2*	rs4696480, rs1898830, rs3804099	Significantly associated with asthma susceptibility	(37, 51)
	rs3804100	T allele is significantly associated with allergic asthma	(50)
*TLR3*	-299698G>T, 293391G>A [Leu412Phe]	Associated with the respiratory disease phenotype	(70)
	rs1519309	Associated with asthma susceptibility	(71)
*TLR4*	rs4986791	Correlation with asthma susceptibility, specifically among Asian populations	(88)
	rs4986791	Associated with bronchial asthma risk in Chinese children	(89)
	rs1927911	Modifying the influence of environmental factors on the risk of asthma	(90)
*TLR5*	rs5744168	A dominant-negative genetic polymorphism is associated with decreased symptoms in patients with asthma	(95)
	rs5744174	Increasing the susceptibility to bronchiolitis not caused by RSV	(96)
*TLR6*	rs1039559, rs5743810	Significantly associated with childhood farm exposure	(98, 99)
	Ser249Pro	A weak association with childhood asthma	(100)
*TLR7*	rs179008	Increasing the risk of asthma in preschool-aged children after infant bronchiolitis	(108)
	rsl79009, rs5935436	Significant correlations with pediatric asthma	(109)
	rs179009, rs179010, rs1634322,	Associated with eosinophil counts only in male asthmatics	(110)
*TLR8*	rs2159377, rs17256081, rs4830805	Associated with asthma-related phenotypes, including eosinophil counts, serum immunoglobulin E levels, lung function, and asthma severity	(110)
*TLR9*	rs187084	The CC genotype of TLR9 is associated with better asthma control and post-bronchiolitis wheezing	(116)
	rsl87084, rs5743836	Significant correlations with pediatric asthma	(109)
*TLR10*	rs4129009	Increasing the risk of persistent asthma continuing from five to seven years of age until 11–13 years of age	(38, 108)
	rs11096956	The GG genotype is associated with better asthma control and better cardiac function	(116)

The presented frequency for this single nucleotide polymorphism (SNP) is from the original paper.
